# Supplementary material for: Tick hemocytes have a pleiotropic role in microbial infection and arthropod fitness
Source: Nat Commun. 2024 Mar 8;15:2117. doi: 10.1038/s41467-024-46494-3 (PMC10923820; doi:10.1038/s41467-024-46494-3)
Supplement: Supplementary file 1 — Supplementary Information [file 41467_2024_46494_MOESM1_ESM.pdf]

Supplementary Information

Tick hemocytes have a pleiotropic role in microbial infection and arthropod fitness

Agustin Rolandelli[1], Hanna J. Laukaitis-Yousey[1], Haikel N. Bogale[2†], Nisha Singh[1α], Sourabh Samaddar[1], Anya J. O'Neal[1‡], Camila R. Ferraz[1], Matthew Butnaru[3,4], Enzo Mameli[3,5], Baolong Xia[3], M. Tays Mendes[1], L. Rainer Butler[1#], Liron Marnin[1], Francy E. Cabrera Paz[1], Luisa M. Valencia[1], Vipin S. Rana[6], Ciaran Skerry[1], Utpal Pal[6], Stephanie E. Mohr[3], Norbert Perrimon[3,4], David Serre[1,2] and Joao H.F. Pedra[1]*

[1]Department of Microbiology and Immunology, University of Maryland School of Medicine; Baltimore, Maryland, USA.

[2]Institute for Genome Sciences, University of Maryland School of Medicine; Baltimore, Maryland, USA.

[3]Department of Genetics, Blavatnik Institute, Harvard Medical School; Boston, Massachusetts, USA.

[4]Howard Hughes Medical Institute; Chevy Chase, Maryland, USA.

[5]Department of Microbiology, National Emerging Infectious Diseases Laboratories, Boston University School of Medicine; Boston, Massachusetts, USA.

[6]Department of Veterinary Medicine, University of Maryland; College Park, Maryland, USA.

[†]Present address: Rancho BioSciences, San Diego, California, USA.

[α]Present address: Department of Biotechnology, School of Energy Technology, Pandit Deendayal Energy University; Knowledge Corridor, Gandhinagar, Gujarat, India

‡Present address: Immunology Program, Memorial Sloan Kettering Cancer Center; New York, New York, USA.

#Present address: Department of Genetics, Blavatnik Institute, Harvard Medical School; Boston, Massachusetts, USA.

*Correspondence: jpedra@som.umaryland.edu

This file includes:

Supplementary Figures 1-20

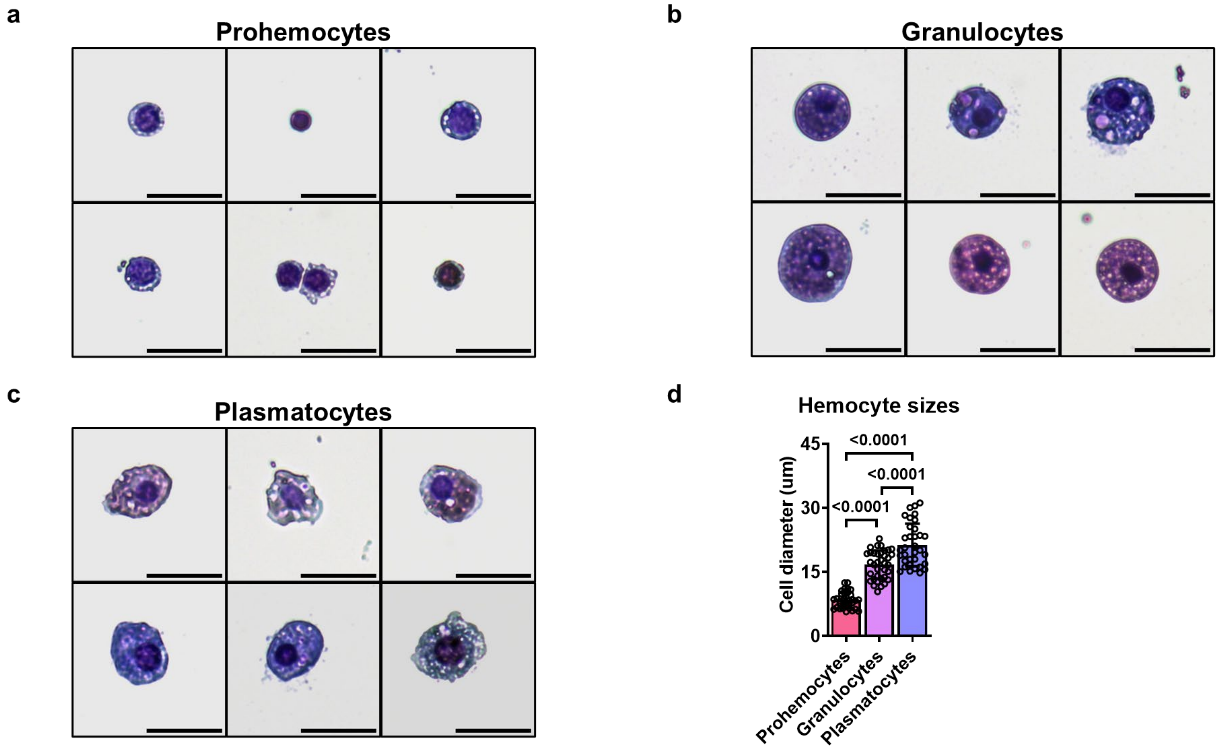

Supplementary Fig. 1. Hemocyte morphotypes observed in *I. scapularis* nymphs. Hemolymph from *I. scapularis* nymphs revealed three morphological types of hemocytes via bright-field microscopy post-staining: **(a)** Prohemocytes, **(b)** Granulocytes and **(c)** Plasmatocytes. Six representative images are provided for each morphotype. Black scale bars indicate a length of 25 µm. **(d)** Quantification of cell diameter (µm) for Prohemocytes (red), Granulocytes (violet) and Plasmatocytes (blue) present in the hemolymph of *I. scapularis* nymphs (*n*=33, 36 and 34). Results are represented as mean ± SD. Three independent experiments were performed. Statistical significance was evaluated by Brown-Forsythe ANOVA test, and significant *p* values (<0.05) are displayed in the figure. Source data are provided as a Source Data file.

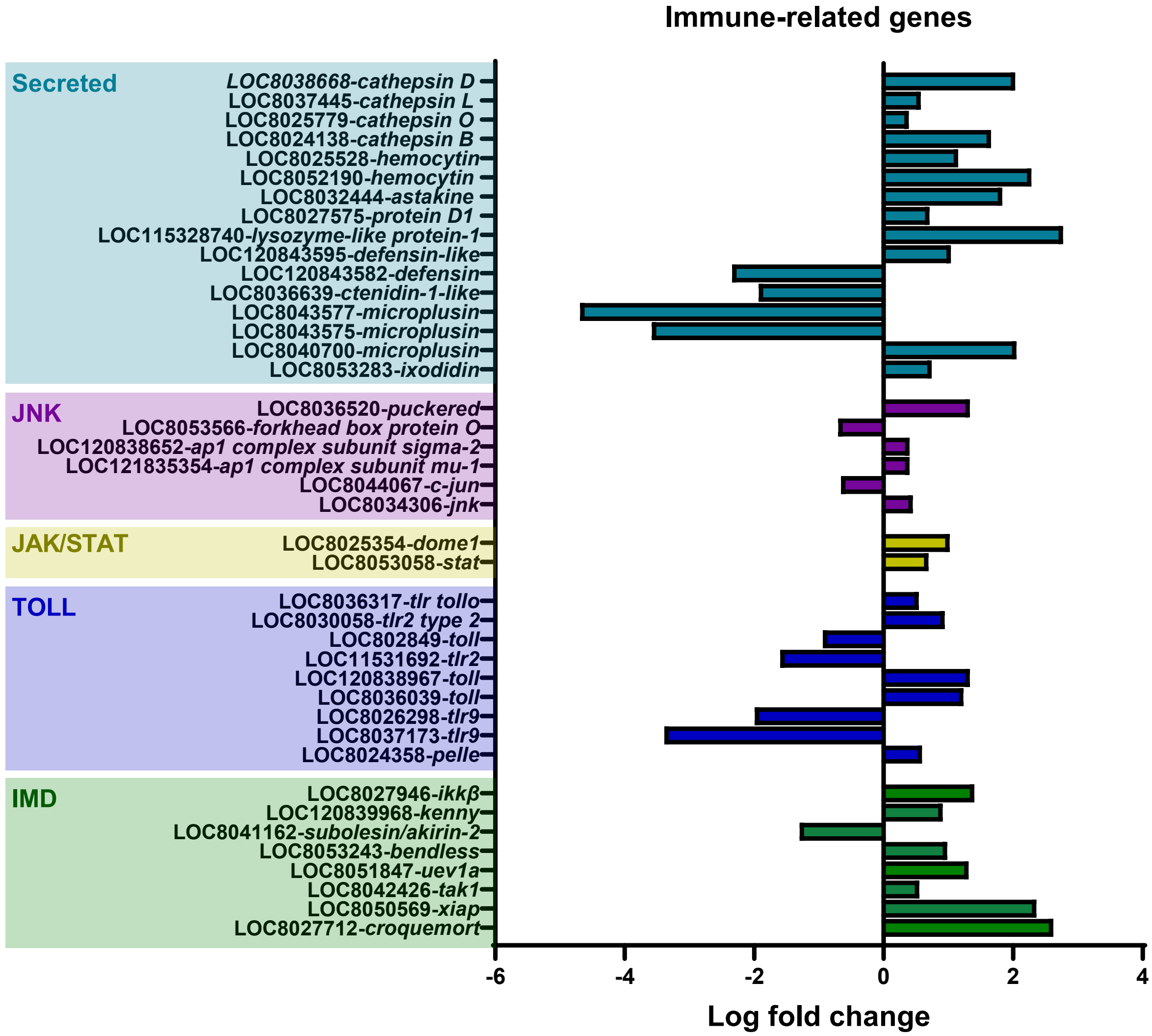

Supplementary Fig. 2. Expression profile of immune genes in hemocyte-enriched samples originated from *I. scapularis* nymphs. Bar graphs depict the log fold change in expression levels of immune-related genes identified from bulk RNA-seq analysis of hemocyte-enriched samples collected from engorged ticks relative to unfed ticks. Genes are color-coded according to their respective pathways or functions. Source data are provided as a Source Data file.

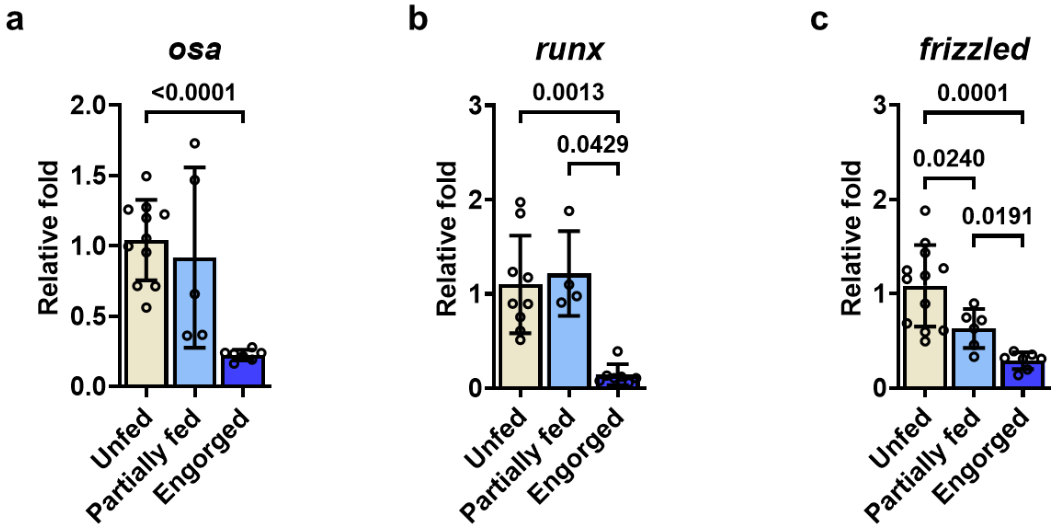

Supplementary Fig. 3. Genes downregulated in hemocyte-enriched samples during feeding. The expression level of **(a)** *osa* (*n*=11, 5 and 7), **(b)** *runx* (*n*=9, 4 and 7) and **(c)** *frizzled* (*n*=12, 6 and 7) was evaluated in hemocyte-enriched samples from unfed (ivory), partially fed (light blue) and engorged (dark blue) ticks by RT-qPCR (samples comprising 40-80 pooled ticks). Results represent mean ± SD. A minimum of 6 independent experiments were performed. Statistical significance was evaluated by Brown-Forsythe ANOVA test, and significant *p* values (<0.05) are displayed in the figure. Source data are provided as a Source Data file. *osa* = *Brahma chromatin remodeling complex subunit osa*; *runx* = *runt-related transcription factor 1*; *frizzled* = *frizzled-5*.

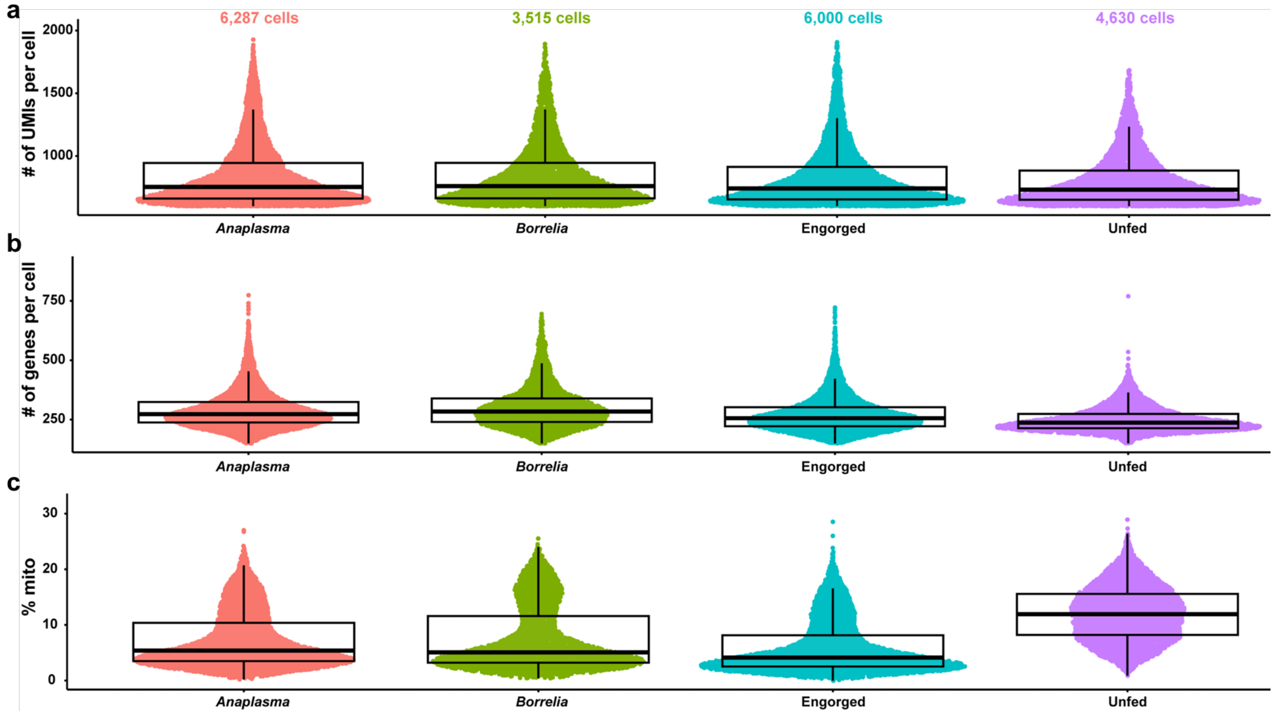

Supplementary Fig. 4. Metrics of sequenced cells present in the hemolymph of *I. scapularis* nymphs. Distribution and median values for **(a)** the number (#) of unique molecular identifiers (UMIs), **(b)** genes detected and **(c)** percentage of mitochondrial transcripts (% mito) per cell in unfed (violet), uninfected engorged (teal), *A. phagocytophilum*-infected (pink) and *B. burgdorferi*-infected (green) *I. scapularis*. Cells of low quality were excluded based on the following criteria: fewer than 600 unique reads, fewer than 150 expressed genes, or mitochondrial transcripts accounting for 30% or more of total transcripts. The total cell count after filtration for each condition is indicated at the top of the graph.

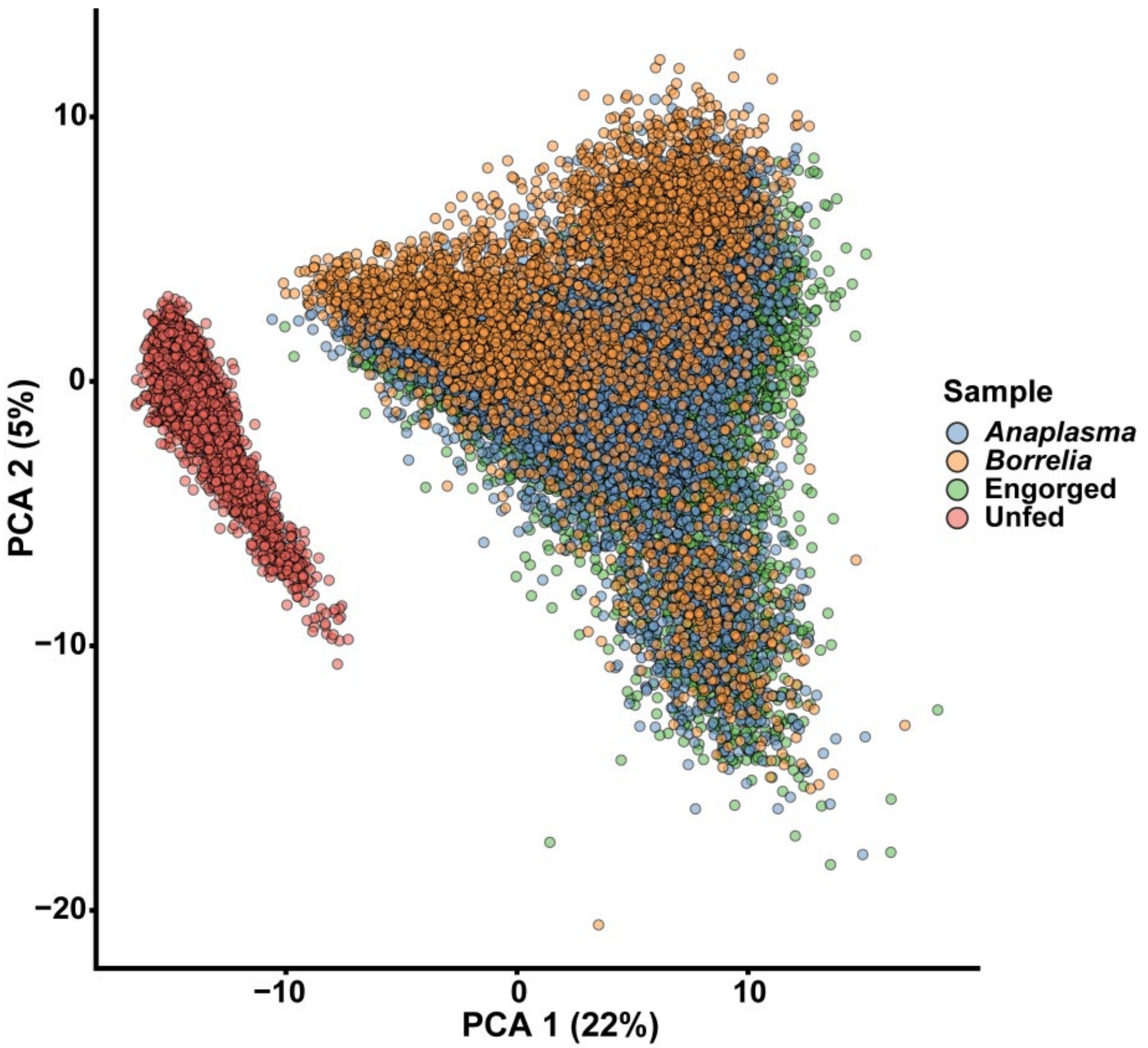

Supplementary Fig. 5. Principal component analysis (PCA) of sequenced cells from the hemolymph of *I. scapularis* nymphs. The PCA plot illustrates the variability among hemocyte-enriched samples derived from unfed (red; 4,630 cells), uninfected engorged (green; 6,000 cells), *A. phagocytophilum*-infected (blue; 6,287 cells) and *B. burgdorferi*-infected (orange; 3,515 cells) ticks.

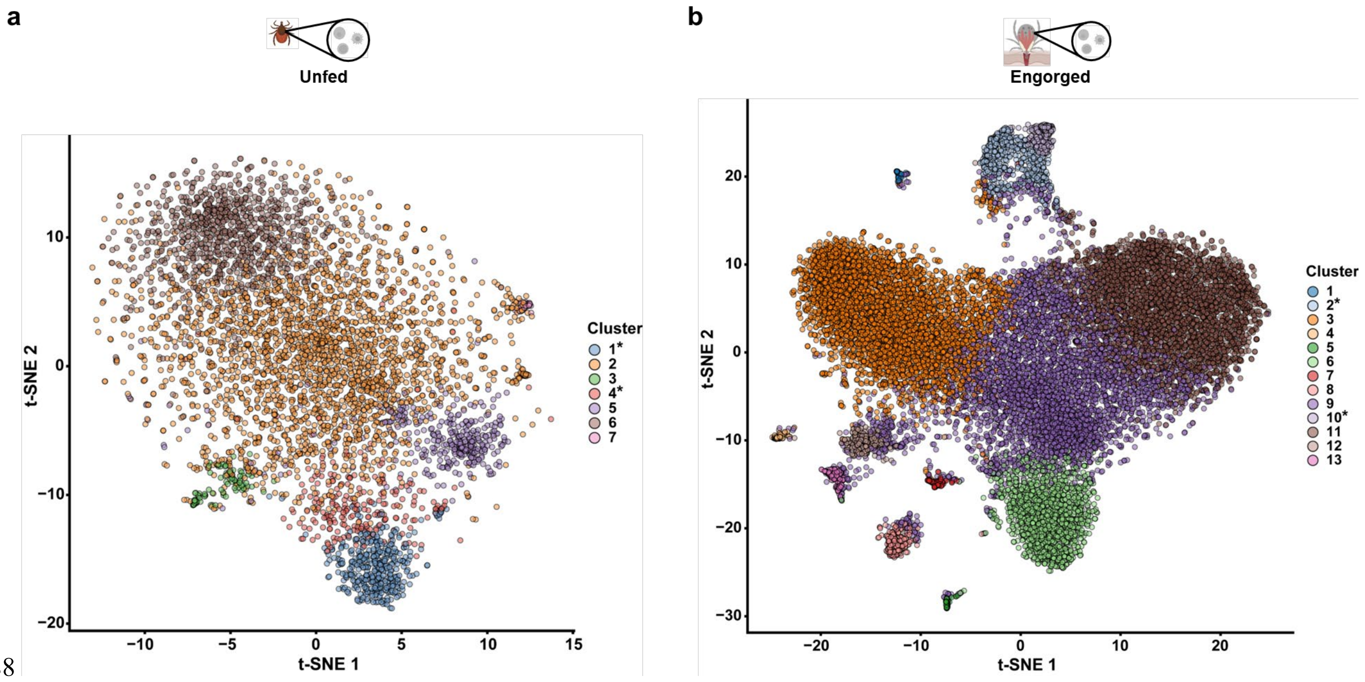

Supplementary Fig. 6. t-distributed stochastic neighbor embedding (t-SNE) plot illustrating cells from the hemolymph of either unfed or engorged *I. scapularis* nymphs. (a) Represents the unfed condition with 4,630 cells, while **(b)** depicts the engorged condition comprising 15,802 cells. The engorged dataset contains hemocyte-enriched samples from uninfected (6,000 cells), *A. phagocytophilum*-infected (6,287 cells) and *B. burgdorferi*-infected (3,515 cells) ticks. Asterisks denote clusters that were subsequently combined due to similarities in marker gene profiles (refer to Supplementary Data 3-4). Tick images in **(a-b)** were created with BioRender.com.

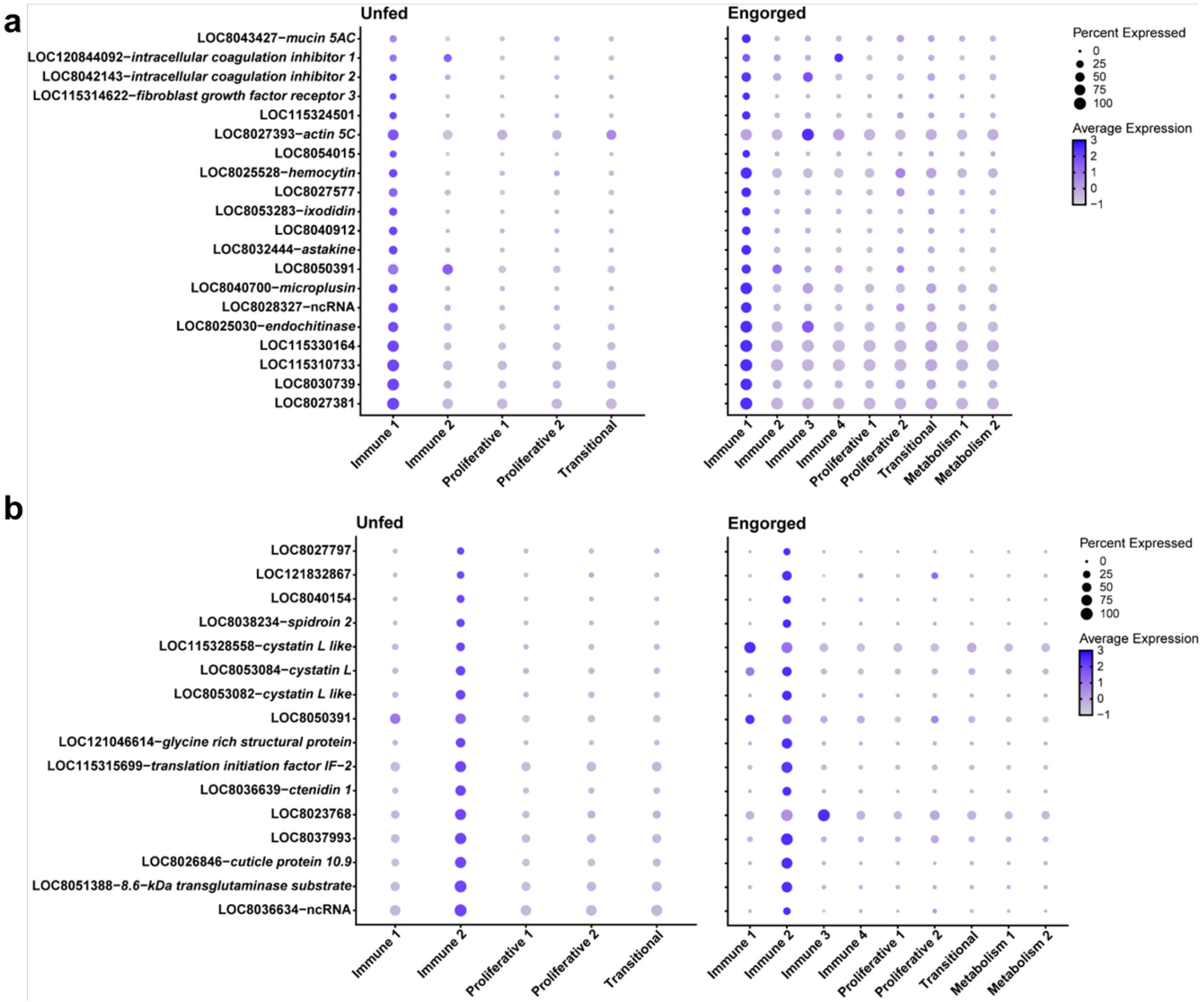

Supplementary Fig. 7. Dot plots comparing the expression of marker genes associated with immune clusters between unfed and engorged ticks. Marker genes for the **(a)** Immune 1 and **(b)** Immune 2 hemocyte clusters are displayed for the unfed (left) and engorged (right) conditions. The plot showcases the top 20 common marker genes across both conditions. Color intensity demarks gene expression level, while the size of the dot indicates the percentage of cells within individual clusters expressing the corresponding gene.

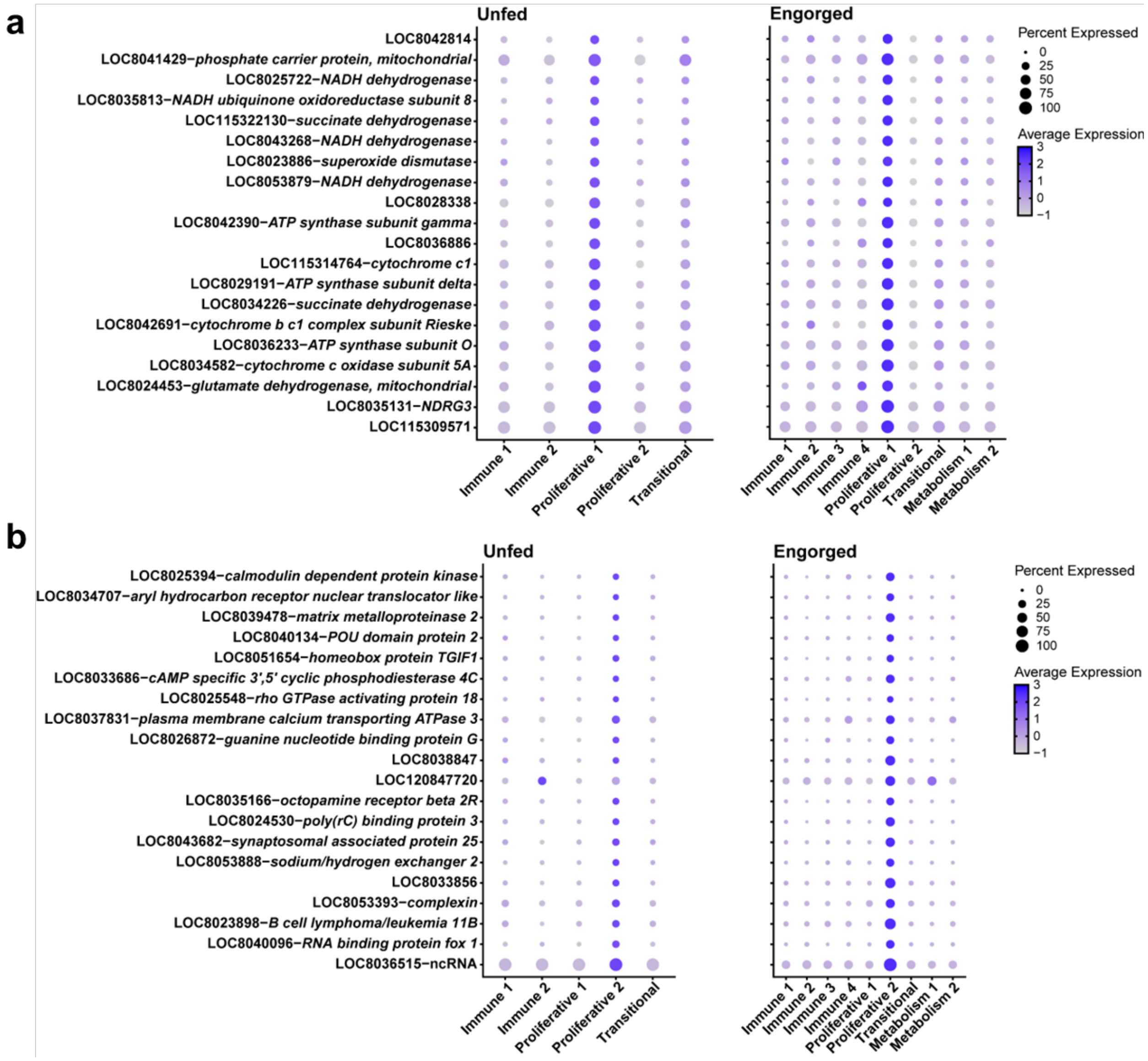

Supplementary Fig. 8. Dot plots comparing the expression of marker genes associated with proliferative clusters between unfed and engorged ticks. Marker genes for the **(a)** Proliferative 1 and **(b)** Proliferative 2 hemocyte clusters are displayed for the unfed (left) and engorged (right) conditions. The plot showcases the top 20 common marker genes across both conditions. Color intensity demarks gene expression level, while the size of the dot indicates the percentage of cells within individual clusters expressing the corresponding gene.

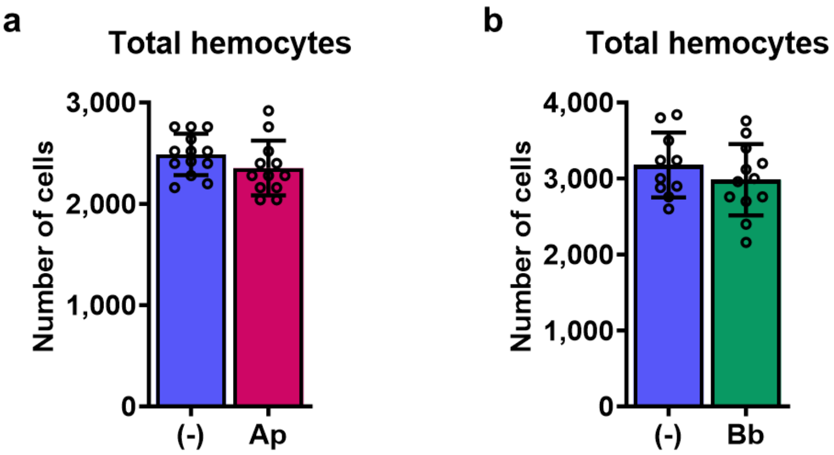

Supplementary Fig. 9. Comparative analysis of total hemocytes between infected and uninfected ticks. Hemocytes from *I. scapularis* nymphs that fed on **(a)** *A. phagocytophilum*- (pink, *n*=13 and 12) or **(b)** *B. burgdorferi*-infected (green, *n*=10 and 12) mice compared to those from uninfected controls. Results represent mean ± SD. A minimum of two independent experiments were performed. Statistical significance was evaluated by an unpaired two-tailed t-test with Welch's correction. Source data are provided as a Source Data file. (-) = Uninfected; Ap = *A. phagocytophilum*; Bb = *B. burgdorferi*.

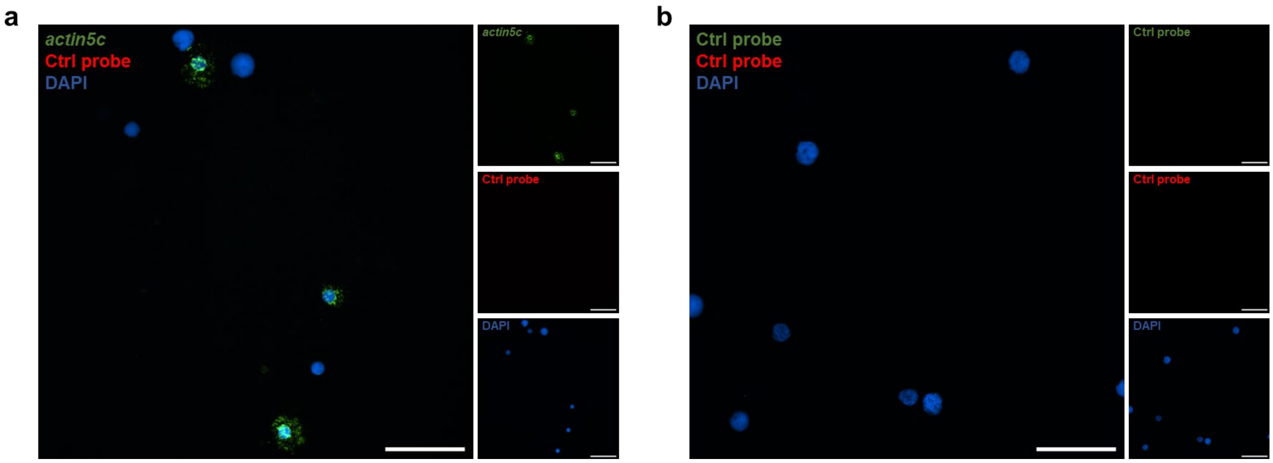

Supplementary Fig. 10. RNA FISH of hemocyte-enriched samples from *I. scapularis* nymphs. (a) RNA FISH images of cells from the hemolymph of *I. scapularis,* labeled for *actin5c* (positive control; green), an irrelevant gene, *gfp* (control probe; red), and nuclei (DAPI; blue). **(b)** RNA FISH images of cells from the hemolymph of *I. scapularis*, probed for an irrelevant gene, *gfp* (control probe; green and red) and nuclei (DAPI; blue), serving as a negative control. White scale bars indicate a length of 50 µm. *gfp* = *green fluorescent protein*; Ctrl probe = control probe.

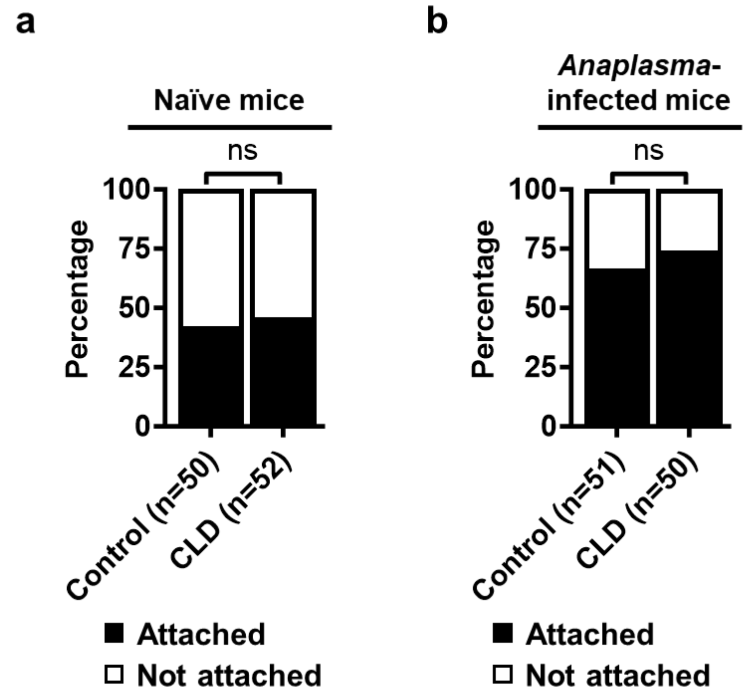

Supplementary Fig. 11. Attachment behavior in clodronate microinjected ticks. Graphs representing the attachment rates of *I. scapularis* nymphs microinjected with clodronate (CLD) or empty liposomes (Control) and subsequently fed on either **(a)** uninfected (naïve) or **(b)** *A. phagocytophilum*-infected mice. Results are displayed as percentage from total number of ticks placed. A minimum of two independent experiments were performed. Statistical significance was evaluated by a Fisher exact test. Source data are provided as a Source Data file. ns = not significant.

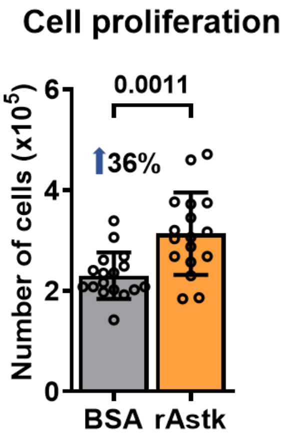

Supplementary Fig. 12. Impact of recombinant Astakine (rAstk) on IDE12 cell proliferation. IDE12 cells ($1x10^5$) were treated with either 0.05 μg/mL of rAstk or BSA (control) for 5 days, after which the total number of live cells were counted (*n*=17 and 17). Results are represented as mean ± SD. Three independent experiments were performed. Statistical significance was evaluated by an unpaired two-tailed *t* test with Welch's correction, and significant *p* values (<0.05) are displayed in the figure. Source data are provided as a Source Data file. rAstk = recombinant Astakine; BSA = Bovine Serum Albumin.

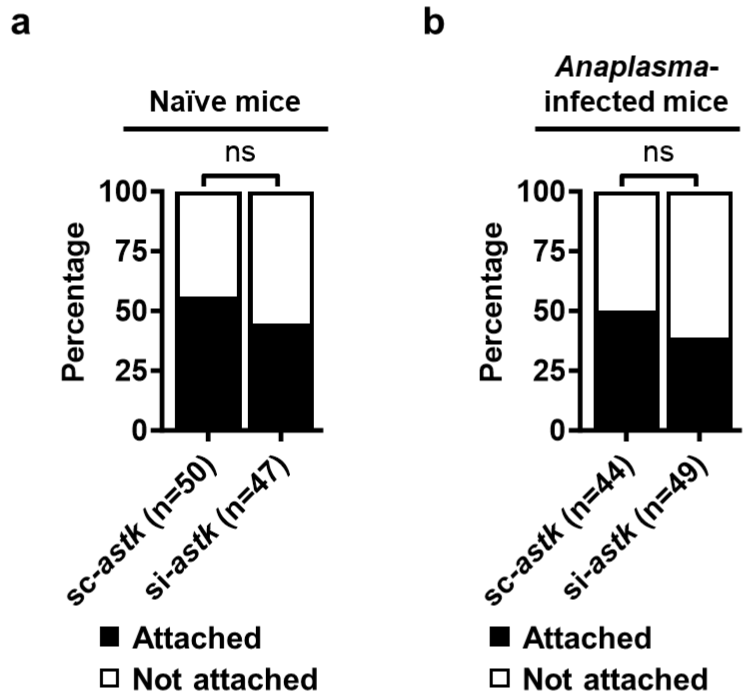

Supplementary Fig. 13. Attachment behavior in *astakine* siRNA microinjected ticks. Graphs representing the attachment rates of *I. scapularis* nymphs microinjected with *astakine* siRNA (si-*astk*) or scrambled RNA (sc-*astk*) and subsequently fed on either **(a)** uninfected (naïve) or **(b)** *A. phagocytophilum*-infected mice. Results are displayed as percentage from total number of ticks placed. A minimum of two independent experiments were performed. Statistical significance was evaluated by a Fisher exact test. Source data are provided as a Source Data file. ns = not significant.

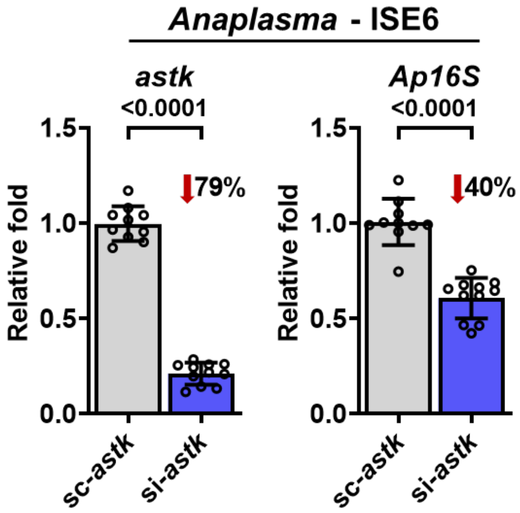

Supplementary Fig. 14. Impact of *astakine* silencing on *A. phagocytophilum* load in tick cells. ISE6 cells (3x10^5) were transfected with either *astakine* siRNA (si-*astk*; blue) or scrambled RNA (sc-*astk*; grey) for seven days and subsequently infected with *A. phagocytophilum* for 48 hours. The efficiency of *astk* silencing (left; n=10 and 11) and *A. phagocytophilum* burden (right; n=10 and 11) are depicted. Bacterial quantification was based on the expression of *A. phagocytophilum 16s rRNA* (*Ap16S*) gene. Results are represented as mean ± SD. Two independent experiments were performed. Statistical significance was evaluated by an unpaired two-tailed t-test with Welch's correction, and significant p values (<0.05) are displayed in the figure. Source data are provided as a Source Data file.

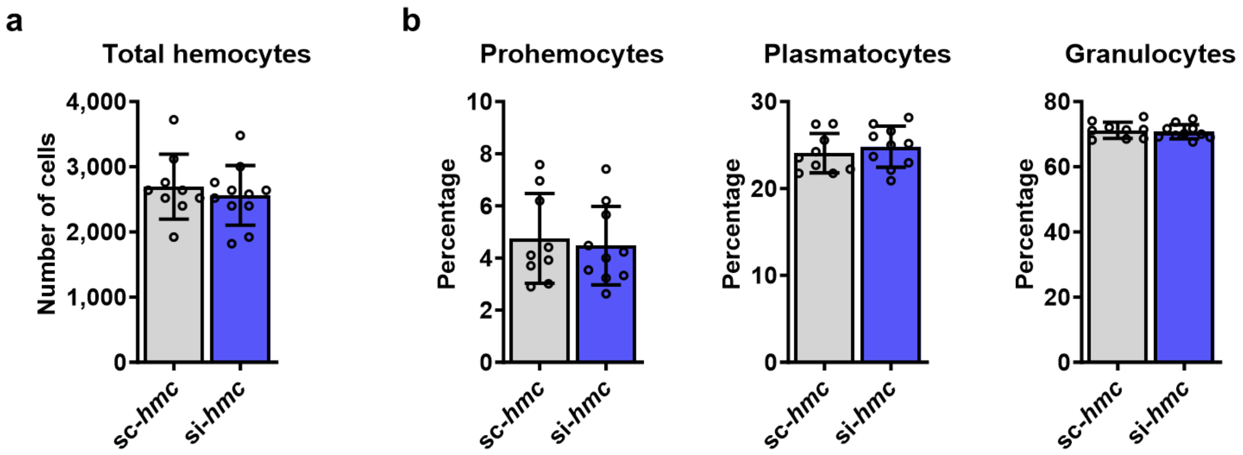

Supplementary Fig. 15. Hemocyte counts in *hemocytin*-silenced ticks. (a) Assessment of total hemocyte counts (*n*=9 and 11) and **(b)** morphotype percentages (prohemocytes, plasmatocytes and granulocytes; *n*=9-11 for all cases) in the hemolymph of ticks microinjected with either *hemocytin* siRNA (si-*hmc*; blue) or scrambled RNA (sc-*hmc*; grey) and fed on uninfected mice. Results are represented as mean ± SD. Two independent experiments were performed. Statistical significance was evaluated by an unpaired two-tailed *t* test with Welch's correction. Source data are provided as a Source Data file.

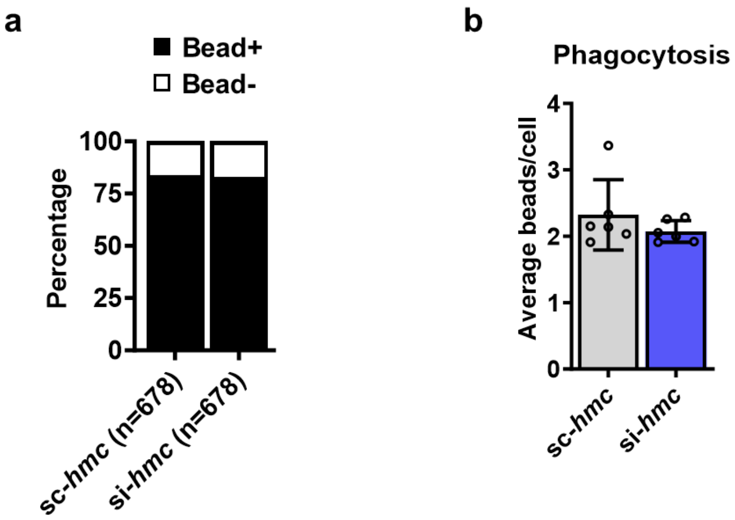

Supplementary Fig. 16. Phagocytosis assays in *hemocytin*-silenced tick cells. IDE12 ($3\text{x}10^5$) cells were transfected with either *hemocytin* siRNA (si-*hmc*) or scrambled RNA (sc-*hmc*) for seven days prior to incubation with fluorescent microspheres for 24 hours. Data were quantified as **(a)** the percentage of cells showing fluorescence from engulfed beads and **(b)** the average number of beads taken up per cell in each field of view. 109-115 cells were analyzed per field (*n*=6 and *n*=6). Data represent two independent experiments. Statistical significance was evaluated by an unpaired *t* test. Source data are provided as a Source Data file. Bead+ = cells positive for beads; Bead- = cells negative for beads.

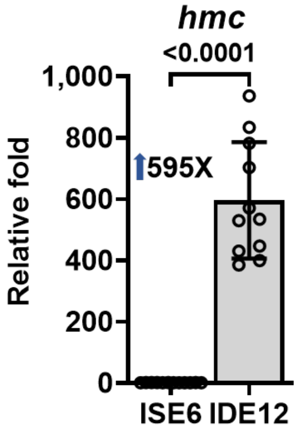

Supplementary Fig. 17. Comparative expression of *hemocytin* in tick cells. The relative expression level of *hemocytin* (*hmc*) in IDE12 cells compared to that in ISE6 cells was determined using RT-qPCR (n=12 and n=11). Results are represented as mean ± SD. Two independent experiments were performed. Statistical significance was evaluated by an unpaired two-tailed t-test with Welch's correction, and significant p values (<0.05) are displayed in the figure. Source data are provided as a Source Data file.

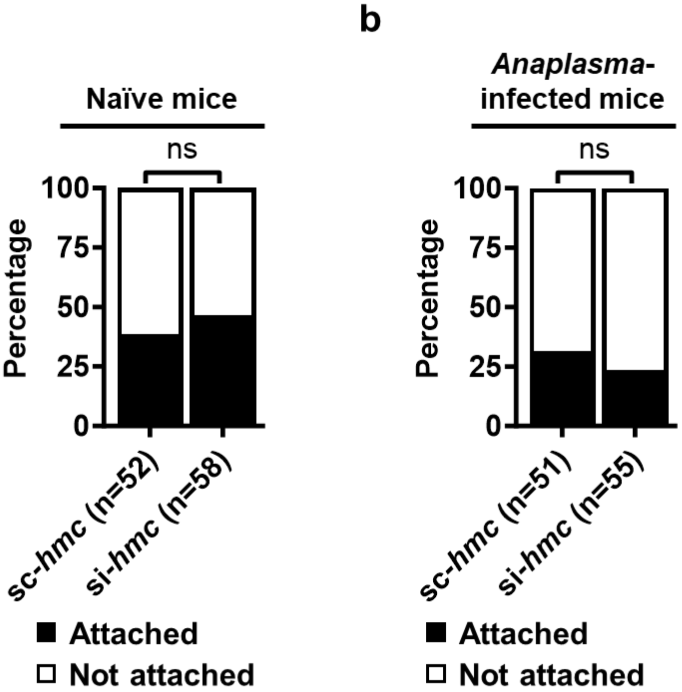

Supplementary Fig. 18. Attachment behavior in *hemocytin* siRNA microinjected ticks. Graphs representing the attachment rates of *I. scapularis* nymphs microinjected with *hemocytin* siRNA (si-*hmc*) or scrambled RNA (sc-*hmc*) and subsequently fed on either **(a)** uninfected or **(b)** *A. phagocytophilum*-infected mice. Results are displayed as percentage from total number of ticks placed. A minimum of two independent experiments were performed. Statistical significance was evaluated by a Fisher exact test. Source data are provided as a Source Data file. ns = not significant.

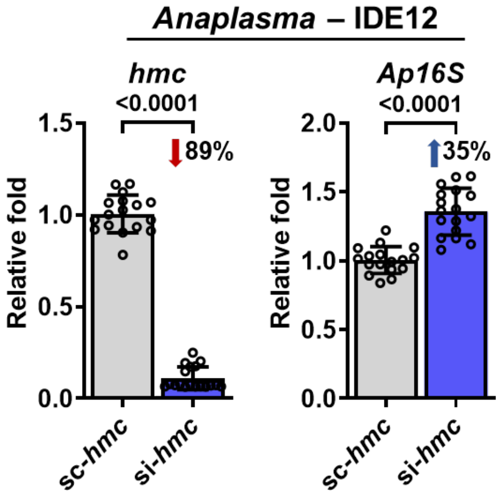

Supplementary Fig. 19. Impact of *hemocytin* silencing on *A. phagocytophilum* load in tick cells. IDE12 cells (3x10^{5}) were transfected with either *hemocytin* siRNA (si-*hmc*; blue) or scrambled RNA (sc-*hmc*; grey) for seven days and subsequently infected with *A. phagocytophilum* for 48 hours. The efficiency of *hmc* silencing (left; *n*=17 and *n*=18) and *A. phagocytophilum* burden (right; *n*=17 and *n*=17) are depicted. Bacterial quantification was based on the expression of *A. phagocytophilum 16s rRNA* (*Ap16S*) gene. Results are represented as mean ± SD. Three independent experiments were performed. Statistical significance was evaluated by an unpaired two-tailed t-test with Welch's correction, and significant *p* values (<0.05) are displayed in the figure. Source data are provided as a Source Data file.

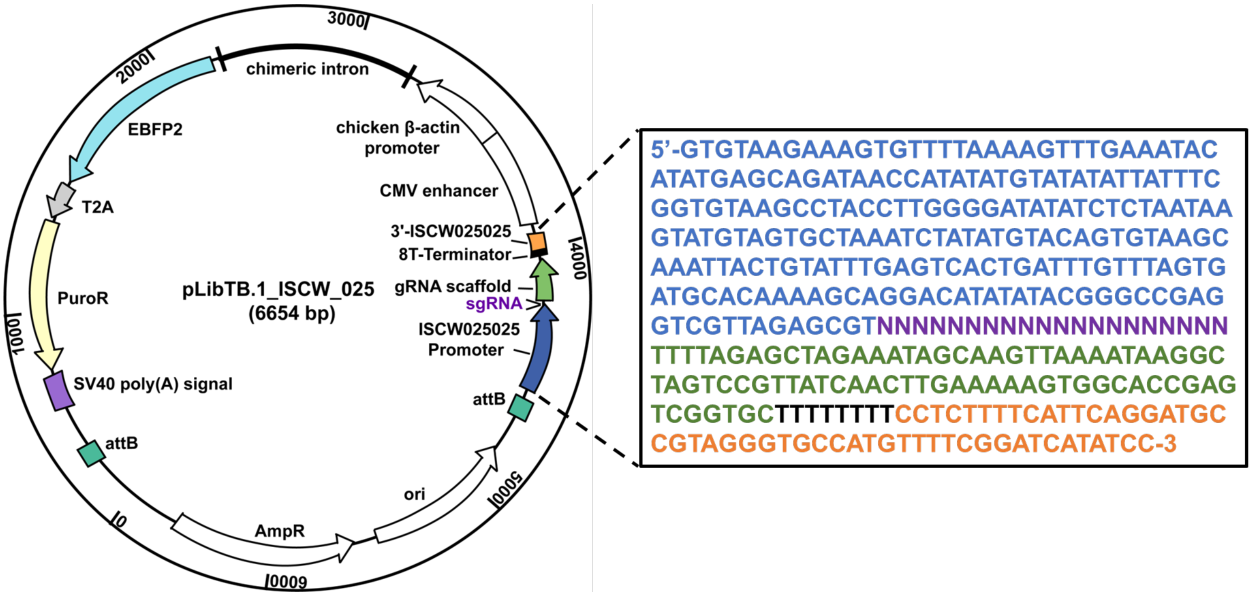

Supplementary Fig. 20. Engineering of the library vector (pLibTB.1_ISCW_025) for single guide RNA expression. (Left) Illustration of the pLibTB.1_ISCW_025 vector, designed for single guide RNA (sgRNA) cloning. The 20 bp sgRNA targeting either the initial exon of the *hemocytin* (*hmc*) gene (ISCP_023268) or a control sequence was cloned between *BbsI* sites. This vector employs the *I. scapularis* U6 promoter for efficient sgRNA expression. (Right) Sequence of the *I. scapularis* U6 promoter and the sgRNA targeting *hemocytin*: *I. scapularis* U6 promoter (ISCW025025; blue); sgRNA spacer for *hmc* or scramble (purple); gRNA scaffold (green); 8-T terminator (black) and U6 snRNA downstream region (3'-ISCW025025; orange). Additional components integrated into the vector include: (1) CAG promoter: Cytomegalovirus (CMV) early enhancer element; promoter, first exon and first intron of the chicken *β-actin* gene and splice acceptor of the rabbit *β-globin* gene; (2) EBFP2: constitutively fluorescent enhanced blue fluorescent protein; (3) T2A: *Thosea asigna* virus ribosome skipping sequence; (4) PuroR: puromycin resistance (puromycin N-acetyl transferase); (5) SV40: Simian virus 40 termination and poly-adenylation sequence; (6) AmpR: ampicillin resistance (TEM-1 β-lactamase); (7) Ori: origin of replication; and (8) attB: phi-C31 attB sites for recombinase-mediated cassette exchange.
