# Supplementary material for: Tick hemocytes have a pleiotropic role in microbial infection and arthropod fitness
Source: Nat Commun. 2024 Mar 8;15:2117. doi: 10.1038/s41467-024-46494-3 (PMC10923820; doi:10.1038/s41467-024-46494-3)
Supplement: Supplementary file 3 — Description of Additional Supplementary Files [file 41467_2024_46494_MOESM3_ESM.pdf]

Description of Additional Supplementary Files

File Name: Supplementary Data 1
Description: Bulk RNA sequencing results of hemocytes from engorged ticks compared with unfed ticks.

File Name: Supplementary Data 2
Description: Functional enrichment analysis of the differentially expressed genes (DEGs) present in hemocytes from engorged ticks compared with unfed ticks.

File Name: Supplementary Data 3
Description: Marker genes for Cluster 1 and Cluster 4 in hemocytes from unfed ticks.

File Name: Supplementary Data 4
Description: Marker genes for Cluster 2 and Cluster 10 in hemocytes from engorged ticks.

File Name: Supplementary Data 5
Description: Number of cells and frequency of all clusters obtained in the scRNAseq analysis of hemocyte-enriched samples.

File Name: Supplementary Data 6
Description: Complete list of marker genes (FDR < 0.05) for each cluster in unfed ticks.

File Name: Supplementary Data 7
Description: Complete list of marker genes (FDR < 0.05) for each cluster in engorged ticks.

File Name: Supplementary Data 8
Description: Functional enrichment analysis of marker genes present in each cluster of hemocytes from unfed ticks.

File Name: Supplementary Data 9
Description: Functional enrichment analysis of marker genes present in each cluster of hemocytes from engorged ticks.

File Name: Supplementary Data 10
Description: Differentially expressed genes (DEGs, FDR < 0.05) in hemocyte subtypes during *A. phagocytophilum* infection compared to uninfected, and differentially expressed genes (DEGs, FDR < 0.05) in hemocyte subtypes during *B. burgdorferi* acquisition compared to uninfected.

File Name: Supplementary Data 11
Description: Expression profile of the top 20 marker genes for the Immune 1 cluster in hemocytes from engorged ticks compared to unfed (from bulk RNA-seq analysis).

File Name: Supplementary Data 12
Description: Primers and siRNA sequences

File Name: Supplementary Data 13
Description: Resources and reagents available
