# Supplementary material for: Tick hemocytes have a pleiotropic role in microbial infection and arthropod fitness
Source: Nat Commun. 2024 Mar 8;15:2117. doi: 10.1038/s41467-024-46494-3 (PMC10923820; doi:10.1038/s41467-024-46494-3)
Supplement: Supplementary file 17 — Reporting Summary [file 41467_2024_46494_MOESM17_ESM.pdf]

nature portfolio

Corresponding author(s): Joao H. F. Pedra

Last updated by author(s): Feb 22, 2024

Reporting Summary

Nature Portfolio wishes to improve the reproducibility of the work that we publish. This form provides structure for consistency and transparency in reporting. For further information on Nature Portfolio policies, see our Editorial Policies and the Editorial Policy Checklist.

Statistics

For all statistical analyses, confirm that the following items are present in the figure legend, table legend, main text, or Methods section.

n/a	Confirmed	
☐	☒	The exact sample size (n) for each experimental group/condition, given as a discrete number and unit of measurement
☐	☒	A statement on whether measurements were taken from distinct samples or whether the same sample was measured repeatedly
☐	☒	The statistical test(s) used AND whether they are one- or two-sided<br>*Only common tests should be described solely by name; describe more complex techniques in the Methods section.*
☐	☒	A description of all covariates tested
☐	☒	A description of any assumptions or corrections, such as tests of normality and adjustment for multiple comparisons
☐	☒	A full description of the statistical parameters including central tendency (e.g. means) or other basic estimates (e.g. regression coefficient) AND variation (e.g. standard deviation) or associated estimates of uncertainty (e.g. confidence intervals)
☐	☒	For null hypothesis testing, the test statistic (e.g. F, t, r) with confidence intervals, effect sizes, degrees of freedom and P value noted<br>*Give P values as exact values whenever suitable.*
☒	☐	For Bayesian analysis, information on the choice of priors and Markov chain Monte Carlo settings
☐	☒	For hierarchical and complex designs, identification of the appropriate level for tests and full reporting of outcomes
☒	☐	Estimates of effect sizes (e.g. Cohen's d, Pearson's r), indicating how they were calculated

Our web collection on statistics for biologists contains articles on many of the points above.

Software and code

Policy information about availability of computer code

Data collection

Carl Zeiss™ Primo Star™ Microscope, Axiocam 305 color camera (Carl Zeiss), ZEN software (Carl Zeiss), Bioanalyzer (Agilent), multiplexed paired end 100bp Illumina NovaSeq 6000 S4 flowcell, R software, 10X Genomics Chromium system, Nikon W-1 spinning disk confocal microscope, EVOS FL Digital Inverted Microscope (Advanced Microscopy Group), ImageJ software (v1.52), TC20™ automated cell counter (Bio-Rad), CFX96 Touch Real-Time PCR Detection System (Bio-rad), ChemiDoc™ MP Imaging System (Biorad), GraphPad PRISM® (version 9.1.2),

Data analysis

Hemocyte morphological evaluation was done using a Carl Zeiss™ Primo Star™ Microscope. Images were acquired using an Axiocam 305 color camera (Carl Zeiss) with the ZEN software (Carl Zeiss), which was used for measuring cell diameters. This microscope was also used to determine infectious inoculum for Anaplasma and Borrelia experiments.

The RNA integrity (RIN) was assessed for each sample using a Eukaryote Total RNA Nano Chip Assay on a Bioanalyzer (Agilent).

Bulk RNA sequencing was performed on a multiplexed paired end 100bp Illumina NovaSeq 6000 S4 flowcell, generating an average of 92 million read pairs per sample. RIN analysis, library preparation and sequencing were completed by Maryland Genomics at the Institute for Genome Sciences, University of Maryland School of Medicine.

Bulk RNA sequencing analysis was done in R. All reads were aligned to the I. scapularis genome (GCF_016920785.2_ASM1692078v2) using HISAT v2.0.4. The generated count matrix was used as input for differential expression analysis with edgeR v3.36.0.

Single-cell RNA sequencing was performed on a 10X Genomics Chromium controller. Four individually barcoded 3'-end scRNA-seq libraries were prepared according to the manufacturer's protocol. Each library was then sequenced on an Illumina high-output sequencer. Reads were then mapped to the I. scapularis genome (GCF_016920785.2_ASM1692078v2) using HISAT version 2.0.4. Out of the mapped reads, "unique

reads" were identified by keeping only one of the reads that had identical 16-mer 10X Genomics barcode, 12-mer unique molecular identifier, and mapped to the same genomic location of the same strand. Finally, the 10X Genomics barcodes were used to separate reads derived from each individual cell and count the number of unique reads mapped to each annotated I. scapularis gene (from transcription start site to 500 bp after the annotated 3'-end). The scRNA-seq libraries were combined into a single dataset using scran v1.26.2. Genes containing "40s_ribosomal" and "60s_ribosomal" were removed from the entire dataset. Low quality cells were removed with the following thresholds: less than 600 unique reads, less than 150 mapped genes, and 30% or higher mitochondrial transcripts. The remaining transcriptomes were normalized by first calculating size factors via scran functions quickCluster and computeSumFactors and computing normalized counts for each cell with logNormCounts function in scater v1.22.0. For downstream analysis, highly variable genes were selected using getTopHVGs before performing PCA and t-distributed Stochastic Neighbor Embedding (t-SNE) projections. Clustering was conducted using a kmeans value of 20. Differential gene expression between clusters was calculated using the find marker function in scran v1.26.2. The R package slingshot v2.2.1 was used to perform pseudotime inference where trajectories. To determine differences in gene expression between each infected condition compared to the reference factor (uninfected), clusters were grouped based on annotated function (e.g. metabolism, proliferation, immune) and MAST v1.24.1 was used to test for significance under the Hurdle model adjusting for the cellular detection rate. The codes for the scRNA sequence analysis are available through the GitHub websites: https://github.com/Haikelnb/scRNASeq_Analysis and https://github.com/HLaukaitisJ/PedraLab_hemocyte_scRNAseq.

Slides from RNA Scope experiments were allowed to dry overnight before examined under a Nikon W-1 spinning disk confocal microscope. For imaging, the following laser channels were used: 405 nm (DAPI), 488 nm (GFP, C1) and 561 nm (RFP, C2). RNAScope probes (Advanced Cell Diagnostics) used: hemocytin RNA probe (regions 2778-3632 of XM_042287086.1) conjugated with C1, astakine probe (regions 47-800 of XM_040222953.1) conjugated with C2, actin5c probe (regions 2-1766 of XM_029977298.4) conjugated with C1 as a positive control and gfp probe (regions 12-686 of AF275953.1) conjugated with C1 or C2 as negative controls.

For phagocytosis experiments, fluorescence and bright field microscopy images were acquired with an EVOS FL Digital Inverted Microscope (Advanced Microscopy Group) and merged using ImageJ software. Negative and positive cells for fluorescent microspheres and the average number of phagocytosed beads per cell were calculated from the number of green puncta detected in the total cells counted in each field (>100 cells).

Live cell counting was done using the trypan blue exclusion assay with a TC20™ automated cell counter (Bio-Rad).

Gene expression was quantified by qRT-PCR using a CFX96 Touch Real-Time PCR Detection System (Bio-rad).

Western blot images were taken using the ChemiDoc™ MP Imaging System (Biorad) with the Image Lab Software (v5.0, build 18). Densitometry of the acquired images were calculated using ImageJ software (v1.52). Original blots were cropped to show only bands of interest in the main manuscript.

All statistical analysis were done using GraphPad PRISM® (version 9.1.2). Outliers were detected by a Grubbs' Test, using the GraphPad Quick Cals Outlier Calculator. P values of < 0.05 were considered statistically significant.

For manuscripts utilizing custom algorithms or software that are central to the research but not yet described in published literature, software must be made available to editors and reviewers. We strongly encourage code deposition in a community repository (e.g. GitHub). See the Nature Portfolio guidelines for submitting code & software for further information.

Data

Policy information about availability of data

All manuscripts must include a data availability statement. This statement should provide the following information, where applicable:

- Accession codes, unique identifiers, or web links for publicly available datasets
- A description of any restrictions on data availability
- For clinical datasets or third party data, please ensure that the statement adheres to our policy

All sequencing reads for the bulk RNA sequences were deposited into the NCBI Sequence Read Archive under the BioProject accession PRJNA906572. All scRNA sequences were deposited into the NCBI Sequence Read Archive under the BioProject accession PRJNA905678.

Research involving human participants, their data, or biological material

Policy information about studies with human participants or human data. See also policy information about sex, gender (identity/presentation), and sexual orientation and race, ethnicity and racism.

Reporting on sex and gender	N/A
Reporting on race, ethnicity, or other socially relevant groupings	N/A
Population characteristics	N/A
Recruitment	N/A
Ethics oversight	N/A

Note that full information on the approval of the study protocol must also be provided in the manuscript.

Field-specific reporting

Please select the one below that is the best fit for your research. If you are not sure, read the appropriate sections before making your selection.

☒ Life sciences ☐ Behavioural & social sciences ☐ Ecological, evolutionary & environmental sciences

For a reference copy of the document with all sections, see nature.com/documents/nr-reporting-summary-flat.pdf

Life sciences study design

All studies must disclose on these points even when the disclosure is negative.

Sample size	Sample size was determined from relevant literature in the field (doi.org:10.1038/ncomms14401; doi.org:10.1073/pnas.2208673120; doi.org:10.1073/pnas.1808905116; doi.org:10.1126/science.abl3837; doi.org:10.3389/fimmu.2023.1094326) and has been shown in previous studies. No sample size calculation were done prior to performing the experiments. In vitro independent experiments were performed with 4–6 replicates each. In vivo independent experiment involved the use of 20-100 ticks each.
Data exclusions	Outliers detected by a Grubbs' Test using the GraphPad Quick Cals Outlier Calculator were excluded from further analysis. The exclusion criteria were pre-established by the Grubb's test, or the extreme studentized deviant (ESD) method, based on a normal distribution and a test statistic (Z) that is calculated from the most extreme data point. The test statistic corresponds to a p-value (0.05) that represents the likelihood of seeing that outlier assuming the underlying data follows a Gaussian distribution.
Replication	All experiments were performed independently at least twice to ensure reproducibility. All independent replicates were successful.
Randomization	Age matched, six- to ten-week-old male mice were used and randomly assigned to treatment groups. I. scapularis nymphs were randomly distributed among treatment groups within and across independent experiments.
Blinding	Investigators were not blinded. Given that we microinject ticks with different siRNAs or compounds, or use infected or uninfected ticks, that we need to track during placement in mice, and the same investigator is performing all the experiments and data analysis, the blinding process is unfeasible. However, when possible, different investigators performed different biological replicates to ensure reproducibility and consistency of results

Reporting for specific materials, systems and methods

We require information from authors about some types of materials, experimental systems and methods used in many studies. Here, indicate whether each material, system or method listed is relevant to your study. If you are not sure if a list item applies to your research, read the appropriate section before selecting a response.

Materials & experimental systems

n/a	Involved in the study	
☐	☒	Antibodies
☐	☒	Eukaryotic cell lines
☒	☐	Palaeontology and archaeology
☐	☒	Animals and other organisms
☒	☐	Clinical data
☒	☐	Dual use research of concern
☒	☐	Plants

Methods

n/a	Involved in the study	
☒	☐	ChIP-seq
☒	☐	Flow cytometry
☒	☐	MRI-based neuroimaging

Antibodies

Antibodies used	Goat Anti-mouse IgG H+L(HRP) (Elabscience E-AB-1001; 1:3,000)<br>Goat Anti-Rabbit IgG H&L (HRP) (Abcam ab97051; 1:4,000-10,000)<br>Rabbit Anti-Mouse Actin (Millipore Sigma A2103; 1:4,000)<br>Rabbit Phospho-SAPK/JNK (Thr183/Tyr185) Polyclonal Ab (Cell Signaling 4668; 1:1,000)<br>Rabbit JNK Polyclonal Ab (Proteintech 10023-1-AP; 1:1,000)<br>Mouse Anti-I. scapularis Relish monoclonal Ab (GenScript N/A; 1:500)
Validation	All antibodies were previously tested to detect the desired Ixodes scapularis proteins (doi: 10.1073/pnas.2208673120 and https://www.biorxiv.org/content/10.1101/2023.09.08.556855v1).

Eukaryotic cell lines

Policy information about cell lines and Sex and Gender in Research

Cell line source(s)	Ulrike G. Munderloh (University of Minnesota) provided all tick cell lines (IDE12 and ISE6). All lines were derived from

Cell line source(s)	respective tick embryos as described in relevant literature. HL-60 (CCL-240) cells were obtained from ATCC, which were derived from a 36-year-old, white, female with acute promyelocytic leukemia.
Authentication	Cell lines were authenticated by distinct morphology using microscopic analysis.
Mycoplasma contamination	Cells were monitored for Mycoplasma contamination once per month. All samples were Mycoplasma negative.
Commonly misidentified lines (See ICLAC register)	No misidentified cell lines were used in this study.

Animals and other research organisms

Policy information about studies involving animals; ARRIVE guidelines recommended for reporting animal research, and Sex and Gender in Research

Laboratory animals	Age matched (6-10 weeks), male C57/BL6 and C3H/HeJ mice were supplied by Jackson Laboratories or the University of Maryland Veterinary Resources. All experiments were done using C57/BL6 mice, except for the those involving B. burgdorferi infection, in which C3H/HeJ mice were used. Mice were maintained using a 12 light/12 dark cycle with temperatures around 20-23°C and a relative humidity between 30-70% with free access to food and water.<br>Ixodes scapularis ticks at the nymphal stage were used in this study from laboratory strains maintained at Oklahoma State University and University of Minnesota.
Wild animals	The study did not involve wild animals
Reporting on sex	Male mice were used as a blood meal source for all feeding experiments due to differences in grooming behavior compared to females. However, all data was generated from nymphal ticks, which are not a sexually dimorphic life stage.
Field-collected samples	No field collected samples were involved in this study.
Ethics oversight	Mouse breeding, weaning and experiments were performed under guidelines from the NIH (Office of Laboratory Animal Welfare (OLAW) assurance numbers A3200-01) and pre-approved by the Institutional Biosafety (IBC-00002247) and Animal Care and Use (IACUC-0119012) committee of the University of Maryland School of Medicine.

Note that full information on the approval of the study protocol must also be provided in the manuscript.

Plants

Seed stocks	*Report on the source of all seed stocks or other plant material used. If applicable, state the seed stock centre and catalogue number. If plant specimens were collected from the field, describe the collection location, date and sampling procedures.*
Novel plant genotypes	*Describe the methods by which all novel plant genotypes were produced. This includes those generated by transgenic approaches, gene editing, chemical/radiation-based mutagenesis and hybridization. For transgenic lines, describe the transformation method, the number of independent lines analyzed and the generation upon which experiments were performed. For gene-edited lines, describe the editor used, the endogenous sequence targeted for editing, the targeting guide RNA sequence (if applicable) and how the editor was applied.*
Authentication	*Describe any authentication procedures for each seed stock used or novel genotype generated. Describe any experiments used to assess the effect of a mutation and, where applicable, how potential secondary effects (e.g. second site T-DNA insertions, mosiacism, off-target gene editing) were examined.*
